# Prostaglandin E_2_ Increases Lentiviral Vector Transduction Efficiency of Adult Human Hematopoietic Stem and Progenitor Cells

**DOI:** 10.1016/j.ymthe.2017.09.025

**Published:** 2017-10-05

**Authors:** Garrett C. Heffner, Melissa Bonner, Lauryn Christiansen, Francis J. Pierciey, Dakota Campbell, Yegor Smurnyy, Wenliang Zhang, Amanda Hamel, Seema Shaw, Gretchen Lewis, Kendrick A. Goss, Olivia Garijo, Bruce E. Torbett, Holly Horton, Mitchell H. Finer, Philip D. Gregory, Gabor Veres

**Affiliations:** 1 bluebird bio, Inc., 60 Binney Street, Cambridge, MA 02142, USA; 2 Department of Molecular and Experimental Medicine, The Scripps Research Institute, La Jolla, CA 92037, USA

**Keywords:** hematopoietic stem cell, gene therapy, hemoglobinopathy, vector copy number, lentiviral vector, transduction, prostaglandin E_2_

## Abstract

Gene therapy currently in development for hemoglobinopathies utilizes ex vivo lentiviral transduction of CD34^+^ hematopoietic stem and progenitor cells (HSPCs). A small-molecule screen identified prostaglandin E_2_ (PGE_2_) as a positive mediator of lentiviral transduction of CD34^+^ cells. Supplementation with PGE_2_ increased lentiviral vector (LVV) transduction of CD34^+^ cells approximately 2-fold compared to control transduction methods with no effect on cell viability. Transduction efficiency was consistently increased in primary CD34^+^ cells from multiple normal human donors and from patients with β-thalassemia or sickle cell disease. Notably, PGE_2_ increased transduction of repopulating human HSPCs in an immune-deficient (nonobese diabetic/severe combined immunodeficiency/interleukin-2 gamma receptor null [NSG]) xenotransplantation mouse model without evidence of in vivo toxicity, lineage bias, or a de novo bias of lentiviral integration sites. These data suggest that PGE_2_ improves lentiviral transduction and increases vector copy number, therefore resulting in increased transgene expression. As a result, PGE_2_ may be useful in clinical gene therapy applications using lentivirally modified HSPCs.

## Teaser

Heffner et al. performed a small-molecule screen and identified prostaglandin E_2_ (PGE_2_) as a positive mediator of lentiviral transduction of CD34^+^ cells. Their data suggest that PGE_2_-mediated improvements in transduction efficiency may aid in clinical gene therapy applications using lentivirally modified HSPCs.

## Introduction

Hematopoietic stem cell transplantation is a potentially curative therapy for multiple clinical indications. As the only long-term self-renewing cell of the hematopoietic system, long-term hematopoietic stem cells (LT-HSCs) are the optimal targets for gene therapy for patients with non-malignant disorders currently treated with allogeneic stem cell transplant. Early promising results with therapeutic applications of lentiviral vector (LVV)-transduced hematopoietic stem cells (HSCs) have been achieved.^1^, ^2^, ^3^, ^4^, ^5^ Despite these early successes, it has been challenging to achieve robust and reliable genetic modification of HSCs for all patients and across a variety of therapeutic indications.^6^ Overcoming this challenge would expand the therapeutic potential of stem cell-based gene therapy, particularly in disorders where a high level of transgenic expression is required. HSC resistance to infection has been attributed to the quiescent (G_0_) phase of the cell cycle of HSCs^7^ or to other innate immune defenses against viral transduction at the level of viral fusion and entry,^8^ including proteasomal activity.^9^ Consequently, approaches to improve lentiviral transduction of HSCs (CD34^+^ cells) have included soluble factors or gene modulation strategies intended to overcome transduction resistance, including modulation of p21 expression, modulation of mTOR activity, and relief of early capsid-dependent barriers to transduction.^10^, ^11^, ^12^ However, to date, no strategies for increasing LVV transduction efficiency had proven to be sufficiently robust to be brought into the clinic for gene therapy of hematopoietic disorders.

To identify novel clinically applicable small-molecule factors that could improve lentiviral transduction of CD34^+^ cells, we performed a high-throughput small-molecule screen on primary CD34^+^ cells from mobilized peripheral blood from healthy human donors. This screen identified prostaglandin E_2_ (PGE_2_) as a candidate vector copy number enhancer. We determined that PGE_2_ increased the level of lentiviral transgene delivery in ex vivo culture for CD34^+^ cells derived from both healthy human donors and human donors with primary hemoglobinopathies. PGE_2_ also increased gene delivery in nonobese diabetic/severe combined immunodeficiency/interleukin-2 gamma receptor null (NSG)-repopulating cells. Moreover, PGE_2_ did not exhibit bias relative to the integration-site profile in CD34^+^ cells transduced in the absence of PGE_2_. Cumulatively, these data support the potential use of PGE_2_ to increase LVV transduction of HSCs for clinical gene therapy applications.

## Results

### Small-Molecule Screen Identifies Candidate Soluble Factors to Improve Transduction of CD34^+^ Cells

In order to identify candidate molecules that could improve lentiviral transduction of CD34^+^ cells in an ex vivo culture protocol, we performed a small-molecule screen for improved transduction of CD34^+^ cells with a standard vesicular stomatitis virus G (VSVG)-pseudotyped GFP-containing LVV. To facilitate the potential for rapid implementation in a Good Manufacturing Practice process, we selected the ScreenWell US Food and Drug Administration (FDA)-approved Drug Library v2 (Enzo Life Sciences), which contained more than 780 compounds, including known antiretroviral compounds that could serve as negative controls and vehicle-only wells that would serve as no-supplement controls. We prestimulated ∼6 × 10^7^ CD34^+^ cells enriched from mobilized peripheral blood (mPB) from a healthy human subject for 48 hr at 1 × 10^6^ cells/mL in cytokine-supplemented media, followed by transduction with a GFP lentivirus at an MOI of 25 and a distribution of ∼50,000 cells/well in a 96-well format. We then added compounds to a final concentration of 10 μM, each concurrent with lentiviral transduction, and washed after 24 hr of transduction. Cells were then cultured for an additional 72 hr in cytokine-supplemented media, and volumetric flow cytometry analysis was performed to simultaneously measure cell yield and GFP positivity for all 780 compounds.

**Figure 1:**
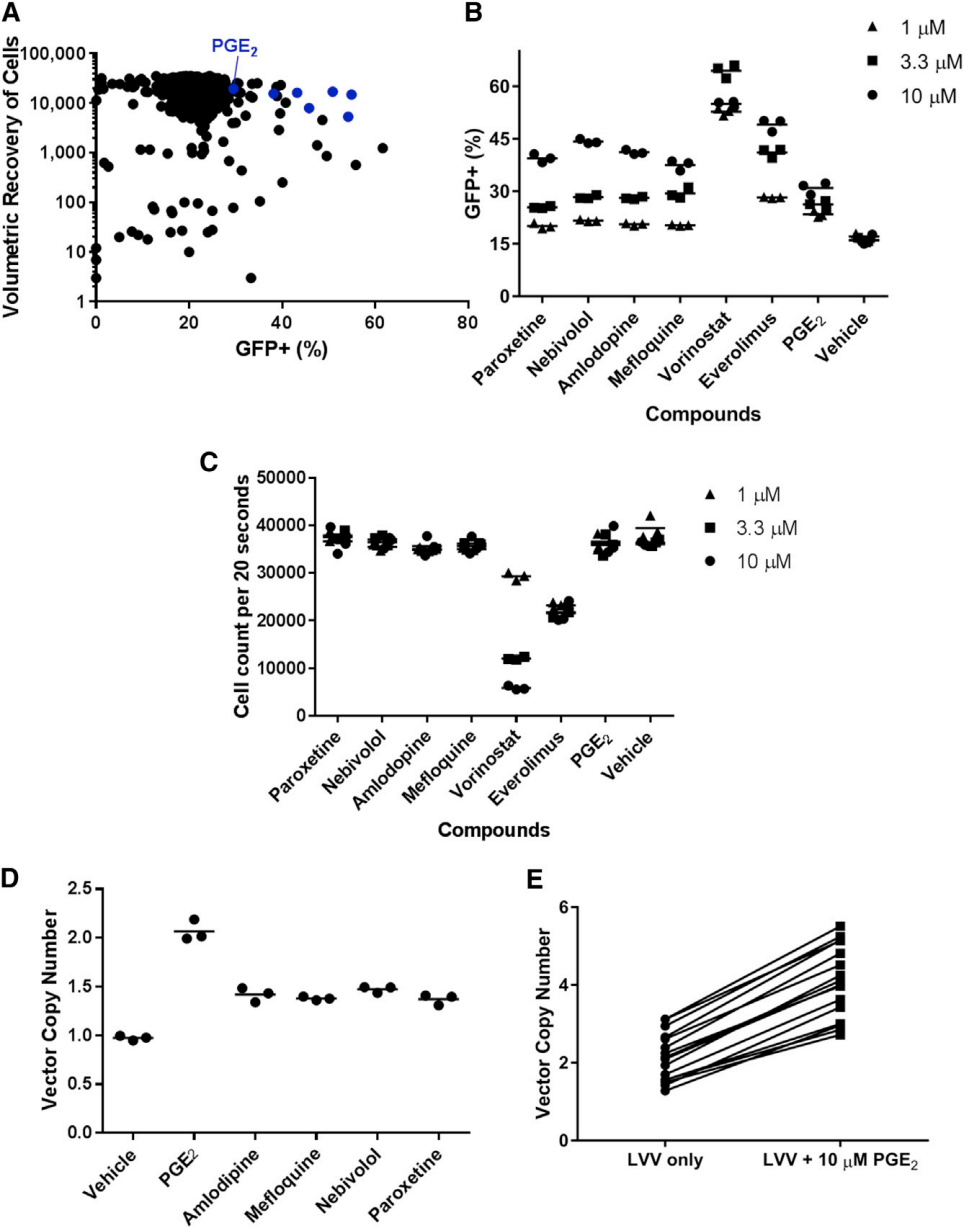
PGE_2_ Enhances Transduction of CD34^+^ Cells with LVV (A) The results of a 780-compound small-molecule screen are depicted. Each compound is represented as a data point with the percentage of GFP^+^ cells indicated on the x axis and the volumetric recovery of cells, on a log scale, indicated on the y axis. Blue dots denote compounds selected for follow-up analysis. (B and C) In a follow-up experiment with 10, 3.3, or 1 μM of seven candidate compounds, the percentage of GFP^+^ cells (B) and the volumetric recovery of cells (C) are indicated for triplicate wells per compound per concentration of compound, as assessed at day 7 post-transduction. (D) Transduction of CD34^+^ cells with BB305 LVV supplemented with 10 μM candidate compound as indicated; the mean VCN is indicated for triplicate wells per compound, as assessed at day 7 post-transduction. (E) Mean VCN from CD34^+^ cells derived from 16 unique healthy donor cell lots, transduced with BB305 LVV supplemented with 10 μM PGE_2_ during the transduction step, as assessed at day 7 post-transduction. Pairwise comparisons of VCN for each cell lot, in the presence or absence of PGE_2_, are indicated by a line. LVV, lentiviral vector; PGE_2_, prostaglandin E2; VCN, vector copy number.

As depicted in Figure 1A, under these conditions the majority of compounds supported transduction levels of approximately 20% GFP^+^, which was indistinguishable from the untreated controls. Consistent with their anticipated role in decreasing lentiviral transduction, known antiretroviral compounds such as efavirenz (1.19%), emtricitabine (0.49%), and zalcitabine (0.06%) yielded significantly decreased levels of GFP^+^ cells in this assay. This screen also identified a number of compounds that drove significantly greater levels of transduction in conjunction with favorable cell yields. These compounds included everolimus (mTOR modulation; 54.9% GFP^+^), vorinostat (histone deacetylase [HDAC] inhibition; 54.2%), nebivolol (β1 receptor blocker; 50.9%), paroxetine (selective serotonin reuptake inhibitor; 45.8%), mefloquine (anti-malarial; 43.2%), amlodipine (calcium channel blocker; 38.1%), and dinoprostone (bioactive lipid, hereafter referred to as PGE_2_; 29.5%). Importantly, identification of everolimus and vorinostat is supported by previous publications, which also identified mTOR inhibition as a modulator of lentiviral transduction.^10^, ^11^, ^13^, ^14^

Based on this primary screen, we selected seven compounds for further analysis. Specifically, we performed an additional transduction of CD34^+^ cells with GFP LVV in the presence of each molecule using a small concentration titration (10, 3, or 1 μM). As illustrated in Figures 1B and 1C, paroxetine, nebivolol, amlodipine, mefloquine, and PGE_2_ yielded elevated GFP^+^ cells relative to vehicle in a concentration-dependent manner, with cell yield equivalent to that of vehicle. By contrast, we were unable to observe cell yield equivalent to that of vehicle in our analysis of everolimus or vorinostat at the tested concentrations, and we therefore eliminated these compounds from further study.

### PGE_2_ Promotes Transduction of CD34^+^ Cells with a Globin-Containing LVV

We then further tested our lead compounds in a preclinical model of LVV-mediated gene therapy for severe hemoglobinopathies. We transduced mPB-derived CD34^+^ cells from healthy human donors using a recombinant LVV encoding the human β-globin gene at an MOI of 25 in the presence or absence of 10 μM of each candidate compound. As illustrated in Figure 1D, we observed the greatest increase in vector copy number (VCN) with 10 μM PGE_2_. Based on these results, we selected PGE_2_ for further study.

To assess the robustness of the transduction enhancement effect of PGE_2_, we transduced mPB CD34^+^ cells from 16 healthy donors with recombinant LVV encoding the human β-globin gene in the presence or absence of 10 μM PGE_2_. As illustrated in Figure 1E, PGE_2_ resulted in improved transduction across 16 lots of CD34^+^ cells from healthy donors, with VCN increasing by an average of 1.9-fold for each donor sample (0 μM PGE_2_: mean VCN 2.14 ± 0.62, range 1.28–3.12; 10 μM PGE_2_: mean VCN 4.08 ± 0.93, range 2.72–5.51). Based on these observations, we conclude that exposure to 10 μM PGE_2_ during LVV transduction of CD34^+^ cells results in an approximately 2-fold increase in gene marking of CD34^+^ HSCs from healthy donors.

To assess the ability of PGE_2_ to increase the effective transduction of CD34^+^ cells over a broader range of MOI, we transduced CD34^+^ cells at MOIs of 4, 8, 12, 16, and 32 in the presence or absence of 10 μM PGE_2_. As illustrated in Figure S1, although we saw a relative “plateau” of transduction at higher MOI in unsupplemented samples, we observed a progressive increase in VCN when increasing MOI in the presence of 10 μM PGE_2_. VCNs achieved upon supplementation with PGE_2_ were, in all cases, elevated relative to CD34^+^ cells transduced in the absence of PGE_2_. We thus conclude that supplementation with PGE_2_ is sufficient to increase transduction levels beyond the range that can be expected to be achieved through modulation of MOI alone.

Finally, to further support the safety profile of exposure to 10 μM PGE_2_ during LVV transduction, we assessed the viability and cell counts of CD34^+^ cells cultured in the presence or absence of 10 μM PGE_2_ during LVV exposure, and compared these results to cells cultured in the absence of LVV. As illustrated in Figure S2, we did not see evidence of toxicity or diminished cell yields associated with PGE_2_ exposure. Thus, based on our aggregate experience with in vitro assays, we conclude that supplementation with 10 μM PGE_2_ during LVV transduction is associated with a favorable safety and efficacy profile, supporting subsequent in vivo studies.

### PGE_2_ Does Not Promote Viral Entry

To determine at which step in the LVV transduction cycle PGE_2_ exerts an effect, we performed a β-lactamase (BlaM) assay using VPR-BlaM-loaded LVV particles that readout viral entry versus later stage of the LVV integration process.^15^ We did not observe an increase in BlaM^+^ cells when CD34^+^ cells were transduced with this LVV in the presence of 10 μM PGE_2_ as compared to cells transduced in the presence of vehicle control (Figure S3). This suggests that PGE_2_ does not exert its transduction enhancement effects via elevated levels of viral entry during LVV transduction of CD34^+^ cells.

### PGE_2_ Exposure during Ex Vivo Transduction Is Associated with Comparable Human CD45^+^ Engraftment and Elevated VCN in NSG Xenotransplant

**Figure 2:**
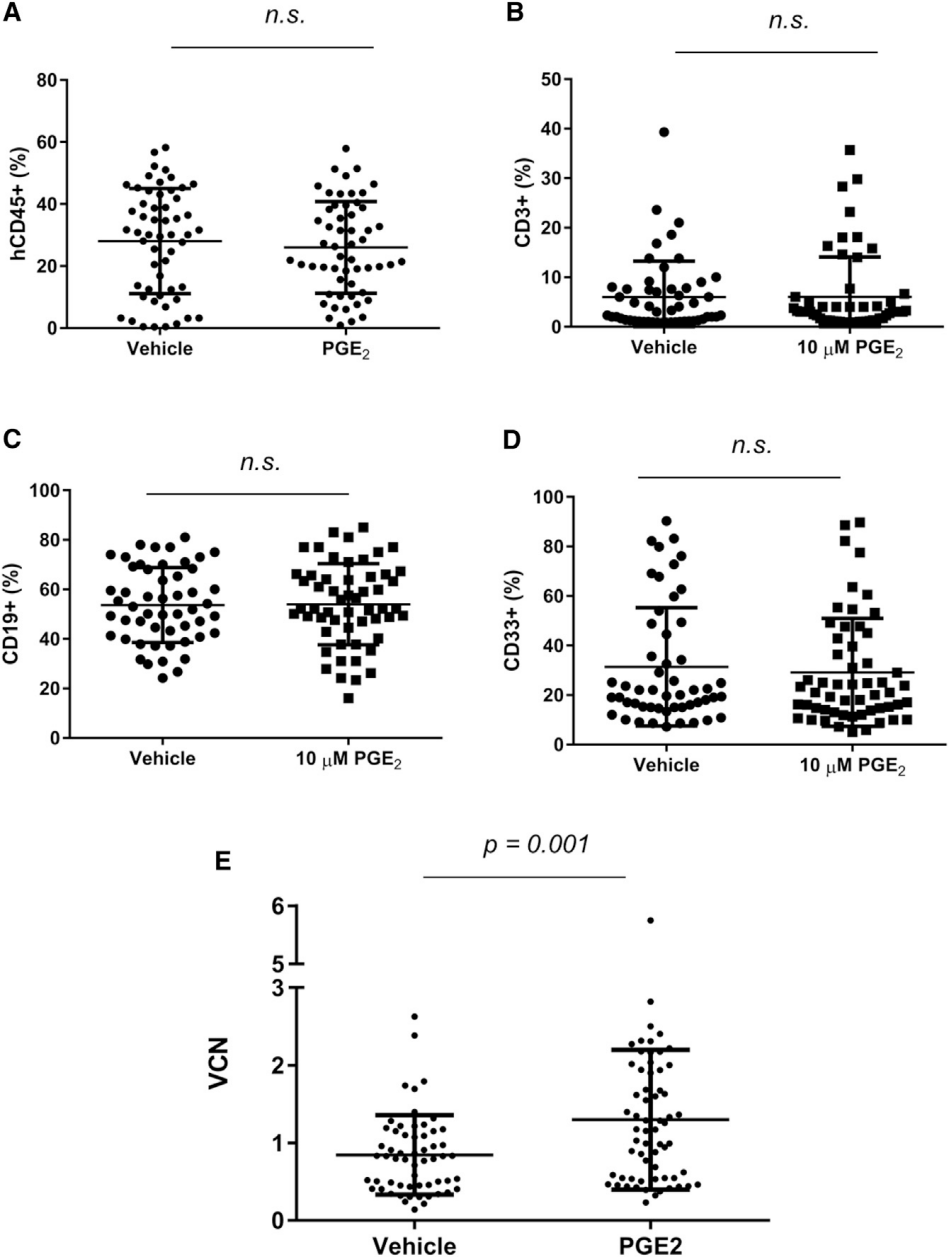
PGE_2_ Exposure during Ex Vivo Transduction Maintains huCD45^+^ Engraftment while Increasing VCN at a 4-Month Time Point in an NSG Xenotransplant Setting (A) Aggregate huCD45^+^ chimerism in bone marrow of engrafted mice at 4 months post-transplant. (B–D) Lineage analysis of huCD45^+^ cells in the bone marrow of engrafted mice at 4 months post-transplant, indicating the frequency of huCD45^+^ cells also positive for CD3 (B), CD19 (C), and CD33^+^ (D). (E) Aggregate mean VCN, in N = 4 experiments, in bone marrow of engrafted mice at 4 months post-transplant; 10–25 mice per group per experiment. p = 0.001 was calculated for (E) using unpaired two-tailed t test. Error bars indicate SD.

To address the ability of PGE_2_ to mediate enhanced gene transfer to a population of cells enriched for LT-HSCs, we first tested the transduction of a prospectively identified primitive CD34^+^ CD38^−^ cell population sorted from mPB from healthy human donors in the presence or absence of 10 μM PGE_2_. As indicated in Figure S4, 10 μM PGE_2_ resulted in elevated VCN in CD34^+^ CD38^−^ cells (0 μM PGE_2_: mean 0.65 ± 0.23 SD; 10 μM PGE_2_: mean 2.12 ± 0.16 SD). This increase was comparable to the VCN elevation noted in a parallel culture of bulk CD34^+^ cells (0 μM PGE_2_: mean 1.02 ± 0.25 SD; 10 μM PGE_2_: mean 2.65 ± 0.67 SD). These results indicate that adding PGE_2_ during transduction can increase VCN in a population of CD34^+^ cells enriched for potential LT-HSC activity. To demonstrate improved gene transfer to the HSC compartment in a xenotransplant setting, we performed a transplant of bulk CD34^+^ cells transduced with LVV in the presence or absence of PGE_2_ into the NSG mouse model. CD34^+^ cells from mPB of four healthy human donors were transduced with a GFP LVV and 1 million cells transplanted per mouse (15–25 mice per group). For each mouse, we assessed the level of human (hu) CD3 (huCD3), huCD19, huCD33, and huCD45 chimerism, GFP^+^ cells within huCD45^+^ cells, and VCN from bone marrow at 4 months post-transplant. As illustrated in Figure 2A, we did not observe a difference in huCD45 chimerism in bone marrow between vehicle-treated CD34^+^ cells and PGE_2_-treated CD34^+^ cells assessed 4 months after transplant. We did not observe a difference in the differentiation potential of these cells based on the analysis of CD3, CD19, or CD33 staining in the bone marrow (Figures 2B–2D). We observed a net increase in VCN in the bone marrow of engrafted mice at 4 months post-transplant (VCN vehicle = 0.84; VCN PGE_2_ = 1.3; p = 0.001; Figure 2E; Table 1). Transplants with three of four donors resulted in a significant increase in VCN at this time point. In addition to these data with GFP-containing LVV, we subsequently demonstrated the ability of PGE_2_ to mediate elevated levels of VCN 4 months after transplant in a xenotransplant setting using an optimized beta-globin-containing LVV (data not shown; unpublished data). In addition, we observed no lineage skewing due to vehicle exposure relative to mock exposure (Figure S5). Together, these data demonstrate that transplant of CD34^+^ cells transduced with LVV generally results in increased in vivo VCN 4 months after transplant in a humanized mouse model.

**Table 1:** Summary of Four Individual Experiments Depicted in Figure 2E

Transplant	VCN		Fold Change	p Value
	Vehicle	PGE_2_		
1	0.42	0.49	1.16	0.2149
2	1.14	1.88	1.64	0.0014
3	1.04	1.45	1.40	0.0018
4	0.39	0.63	1.61	0.0461
Combined	0.84	1.30	1.54	0.0010

PGE_2_, prostaglandin E_2_; VCN, vector copy number; vehicle, 0.1% DMSO.

### Absence of Integration-Site Bias Associated with PGE_2_-Enhanced LVV Transduction of CD34^+^ Cells

**Figure 3:**
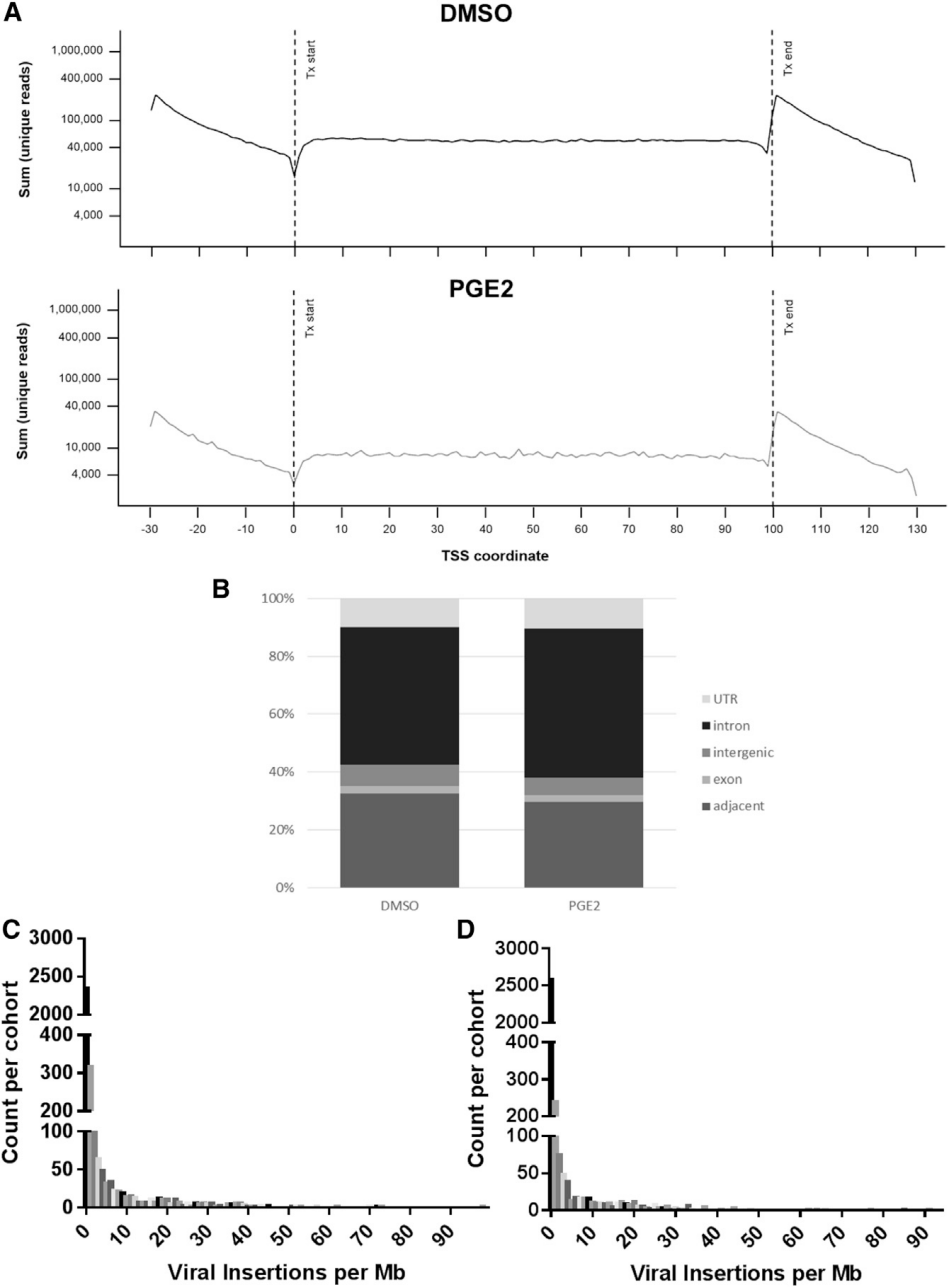
LVV Genomic Integration Profile from Xenotransplanted huCD34^+^ Cells Transduced in the Presence and Absence of PGE_2_ Bone marrow of engrafted mice from Figure 2 at 4 months post-transplant was subjected to LAM-PCR, and libraries were sequenced and aligned to reference human genome. (A) The numbers of unique reads are depicted when occurring within 30 kb upstream of a mapped transcriptional start site, within a genetic region as normalized to the transcriptional start site, or within 30 kb downstream of transcriptional termination sites. (B) Frequency of reads in locations adjacent to transcriptionally active loci, within exons, within intergenic regions, within introns, or within untranslated regions of genes. (C) Number of unique integration sites per megabase of genome, as identified in mice transplanted with cells transduced with LVV in the absence (C) or presence (D) of 10 μM PGE_2_. LAM-PCR, linear amplification-mediated PCR; LVV, lentiviral vector; Mb, megabase; PGE_2_, prostaglandin E_2_; TSS, transcription start site; Tx start, transcriptional start; Tx end, transcriptional end.

To determine whether PGE_2_ modulated the integration-site profile during lentiviral transduction, we performed linear amplification-mediated PCR (LAM-PCR) on post-transplant samples isolated from 4-month bone marrow from 80 engrafted NSG mice, of which 40 had received CD34^+^ cells transduced with a GFP-containing LVV in the presence of 10 μM PGE_2_ and 40 had received CD34^+^ cells transduced in the presence of 0.1% DMSO vehicle control. We first quantified the distribution of integration sites in relation to transcriptional start sites and intragenic regions, as well as downstream of transcriptional stop sites. We observed no difference between PGE_2_-exposed or vehicle-exposed samples in the distribution patterns of integration sites in the genomic regions up to 30 kb upstream or downstream of transcriptional start or termination sites, or in the distribution of integration sites normalized to position within a transcriptional region (Figure 3A). We assessed the integration-site profiles in a broader context in the genome, to assess the distribution patterns in locations adjacent to transcriptionally active loci, within exons, within intergenic regions, within introns, or within untranslated regions of genes. We did not identify differences between PGE_2_- or vehicle-exposed samples in these distribution patterns (Figure 3B). We then identified the most frequent integration sites in each of 80 transplanted mice, and mapped each integration site to a 1-Mb region of the genome. We found no differences between the distribution of the number of insertion sites per megabase in vehicle-treated and PGE_2_-treated cells (Figure 3C). Exposure of CD34^+^ cells to PGE_2_ during LVV transduction was not associated with LVV integration-site bias in 4-month NSG-engrafting LVV-transduced human CD34^+^ cells.

Finally, we addressed the possibility that supplementation with PGE_2_ could influence LVV integration with respect to sites adjacent to known oncogenes or tumor suppressor genes. The lists of tumor suppressor and oncogenes were obtained from UniProt database (PMID 25348405). After completion of integration-site analysis for each engrafted mouse, we annotated each insertion site as being within 30 kb of either oncogene, tumor suppressor, or neither. We calculated the percentages of reads found in tumor suppressor and oncogene loci for each animal and used one-way ANOVA to test for statistical significance between DMSO- and PGE_2_-treated animals. Our data are summarized in Table S1. Interestingly, although we observed a trend toward fewer integration sites adjacent to known oncogene and tumor suppressor genes upon supplementation with PGE_2_ during the transduction process, the trends did not achieve statistical significance (p values were p = 0.87 for oncogenes and p = 0.16 for tumor suppressors). Thus, PGE_2_ does not impact distribution of insertion sites among tumor suppressor or oncogenes. Cumulatively, these analyses demonstrate that PGE_2_ supplementation during LVV transduction is associated with a favorable integration-site safety profile relative to standard LVV transduction processes.

### PGE_2_ Improves In Vitro Transduction of CD34^+^ Cells from People with β-Thalassemia and Sickle Cell Disease

**Figure 4:**
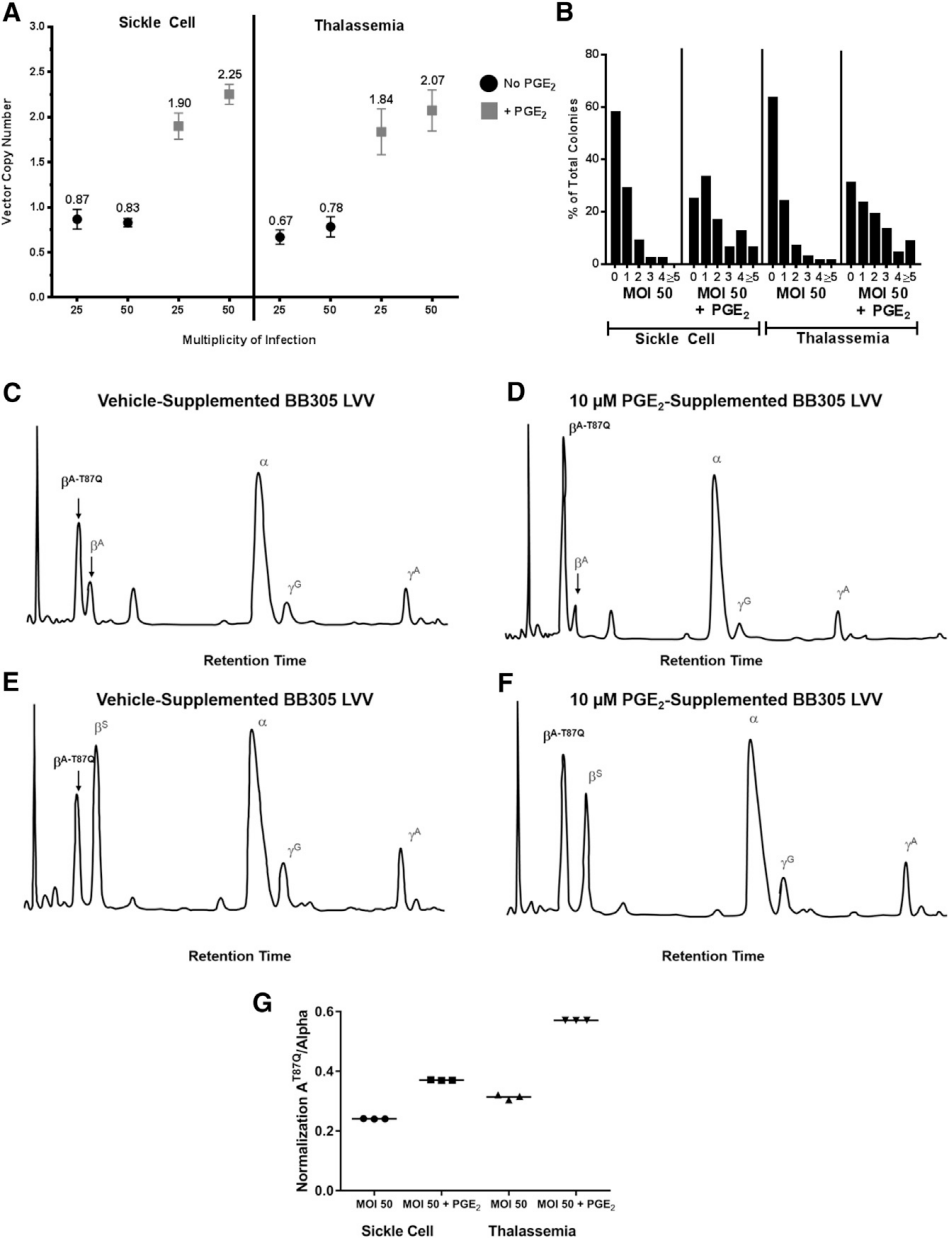
PGE_2_ Improves In Vitro Transduction of CD34^+^ Cells from β-Thalassemia and Sickle Cell Disease Patients (A and B) Mean VCN (A) and distribution of VCN (B) within individual colonies, after methylcellulose culture of primary CD34^+^ cells derived from mPB from a patient with β-thalassemia and from bone marrow from a patient with sickle cell disease, transduced with BB305 LVV, at the indicated MOI, supplemented with 10 μM PGE_2_ or vehicle. Data from (B) are summarized in Table 2. (A) Error bars indicate SD. (C–F) HPLC analysis of pooled BFU-E derived from methylcellulose culture of primary CD34+ cells transduced with BB305 LVV and supplemented with vehicle (C and E) or 10 μM PGE_2_ (D and F). Data from triplicate HPLC samples for (C–F) are summarized in (G). (G) Data from HPLC samples (C–F), performed in triplicate, are summarized. BFU-E, burst-forming unit-erythroid; HPLC, high-performance liquid chromatography; LVV, lentiviral vector; mBP, mobilized peripheral blood; MOI, multiplicity of infection; PGE_2_, prostaglandin E_2_; VCN, vector copy number.

We investigated whether PGE_2_ could promote lentivirally mediated transduction, and increase transgenic hemoglobin protein expression, in primary CD34^+^ cells derived from patients with hemoglobinopathies. CD34^+^ cells from the mPB of a patient with transfusion-dependent β-thalassemia and CD34^+^ cells from the bone marrow of a patient with sickle cell disease were transduced with recombinant LVV encoding the human β-globin gene in the presence or absence of PGE_2_. Following transduction, cells were plated in methylcellulose to assess colony-forming unit potential. We observed an increase in the VCN of pooled colonies transduced in the presence of PGE_2_ relative to vehicle (Figure 4A). Surprisingly, we observed that the VCN in the presence of PGE_2_ during transduction at an MOI of 25 was greater than the VCN in the absence of PGE_2_ at an MOI of 50, suggesting that PGE_2_ can increase LVV transduction of CD34^+^ cells above any increase in VCN from simple increase of the vector MOI used during transduction.

We then performed single-colony analysis of methylcellulose cultures to determine the frequency of transduced myeloid progenitors, as well as the number of integrations per cell. We observed an increase in the total number of vector-containing colonies (a measure of the percentage transduced), as well as an increase in the number of colonies with higher numbers of integration events per cell in both sickle cell disease and thalassemic cells transduced in the presence of 10 μM PGE_2_ relative to control (Figure 4B; Table 2). Finally, we isolated individual and pooled burst-forming unit-erythroid (BFU-E) colonies to perform high-performance liquid chromatography (HPLC) analysis of hemoglobin chains in these cells. We observed elevated levels of vector-derived β-globin in BFU-E derived from CD34^+^ cells transduced with LVV in the presence of 10 μM PGE_2_ in the context of both sickle cell disease and thalassemia CD34^+^ cells (Figures 4C–4G). These data demonstrate that PGE_2_ can promote lentiviral transduction and increases in therapeutic gene expression in primary CD34^+^ cells derived from patients with hemoglobinopathies.

**Table 2:** Single-Colony Analysis of Vector-Containing Colonies (%) and VCN in Sickle Cell Disease and Thalassemic Cells Transduced in the Presence of 10 μM PGE_2_ Relative to Control

	Sickle Cell		Thalassemia	
	MOI 50	MOI 50 + PGE_2_	MOI 50	MOI 50 + PGE_2_
Colonies analyzed (n)	45	48	71	68
Marking (%)	42.2	75.0	36.6	69.1
VCN	0.62	1.75	0.61	1.87

## Discussion

An ongoing challenge to the promise of broad LVV-based gene therapy for hematopoietic indications has been to ensure robust and reliable genetic modification of HSCs. An optimal strategy for clinical application of virally mediated HSC gene transfer would permit delivery of sufficient therapeutic transgene into HSCs, while minimizing potentially adverse integration-site preference of vector in the host genome. Here, we have demonstrated that the addition of PGE_2_ improves lentiviral transduction of human hematopoietic stem and progenitor cells, without evidence of overt integration-site bias in lentivirally transduced cells. We anticipate that PGE_2_ will help low-transducing hematopoietic stem and progenitor cells to achieve better transduction and increase the likelihood of clinical benefit for a broader spectrum of patients. In the context of gene dosing in hemoglobinopathies, and additional indications where gene therapy may require stoichiometric elevations in the level of transgene delivery, improvements in the absolute VCN, mediated by PGE_2_, could be the critical difference ensuring a robust and successful treatment. In addition, even for indications where LVV transduction may already appear sufficient for clinical benefit, the incorporation of PGE_2_ may allow for elevated transduction with less vector and/or reduce patient-to-patient variability in achieving a threshold of transduction. Thus, although our analysis has largely focused on gene therapy for hemoglobinopathies, we anticipate that PGE_2_ could find relevance in many further applications for gene modification in CD34^+^ cells beyond these indications.

PGE_2_ and its chemical relative 16, 16-dimethyl PGE_2_ (dmPGE_2_) have been previously noted to have beneficial effects in promoting self-renewal and transplantation efficacy in hematopoietic stem and progenitor cells (HSPCs).^16^, ^17^ However, the relevance of a salutary effect on HSPC renewal to the goal of increasing viral transduction of HSPCs is not known; in fact, additional candidate HSPC self-renewal factors such as SR1, Wnt3a, SHH, and UM171 were not observed to improve LVV transduction in our hands (data not shown).^18^, ^19^, ^20^, ^21^

Although the full mechanism is still under investigation, our data suggest a role of PGE_2_ that may be unique among the published clinical and preclinical efforts to use PGE_2_ to imbue improved clinical characteristics on CD34^+^ cells. Previous investigators have used short pulses of PGE_2_ to improve the transplantability of cord-blood-derived CD34^+^ cells, or long exposures of PGE_2_ in an attempt to better preserve stem and early progenitor cells during zinc-finger nuclease-mediated cleavage and homology-directed repair with integration-deficient LVV donor template.^22^, ^23^ Because we have not seen transduction enhancement with short pulses (1–2 hr) of PGE_2_ (data not shown) and we observe no effect of PGE_2_ on CD34^+^ cell viability or cell number, we hypothesize that PGE_2_ can exert different mechanisms on CD34^+^ cells depending on the timing or context of exposure.

Interestingly, the marked transduction enhancement effect in vitro was somewhat muted in the transplant setting, potentially because of the general difficulty of transduction of the LT-HSC compartment. Alternatively, perhaps high transduction levels of a non-engrafting cell population aids in over-representing the ex vivo VCN relative to the eventual in vivo VCN of engrafted human cells. Nonetheless, a robust 1.5-fold improvement was observed across donors, which supports consideration of including PGE_2_ in CD34^+^ cell-based gene therapy studies going forward. Of further note, our experience with the transduction-modulating behaviors of the various compounds in this screen help underscores the difficulty in identifying universal “transduction enhancers” for all clinically relevant cell populations and indications. Provided that each compound exerts transduction benefits on bona fide HSCs, a compound that modulates the fraction of corrected cells, may be more important for certain indications than a compound that modulates the VCN per cell. Future studies of the involvement of PGE_2_ in modulating LVV transduction may address potential variability within the HSPC compartment for responsiveness to PGE_2_, for example, through assessment of variable modulation of viral restriction factors among these cell populations, or an assessment of patient-to-patient variability at the HSC level. Although such studies are beyond the scope of this paper, they may further illuminate the challenges in modulating LVV transduction in clinically relevant cell populations.

To our knowledge, we were the first to report that PGE_2_ increases LVV transduction of CD34^+^ cells (Heffner, et al., 2013, Mol Ther., abstract). Indeed, our results are unexpected given that PGE_2_ has been shown to decrease transduction of macrophage lineage cells.^24^, ^25^ Although König et al.^26^ observed that disruption of a prostaglandin synthase, PTGES3, resulted in a diminished level of nuclear import of HIV preintegration complex, Gomez et al.^27^ and Malki et al.^28^ have reported an increased level of nuclear import of protein products in response to PGE_2_ or D_2_ signaling. Future work will address the specific mechanisms whereby PGE_2_ improves LVV transduction of CD34^+^ cells.

## Materials and Methods

### Cell Culture

Research-grade GFP-, globin-, and ALDP-containing LVVs were produced at bluebird bio (Cambridge, MA, USA). CD34-enriched mPB samples from healthy human donors were obtained from AllCells (Emeryville, CA, USA), Key Biologics (Memphis, TN, USA), and primary patients under institutional review board (IRB) approval. PGE_2_, paroxetine, amlodipine, and everolimus were obtained from Cayman Chemical (Ann Arbor, MI, USA). Nebivolol and mefloquine were obtained from Sigma-Aldrich (St. Louis, MO, USA). CD34^+^ cells were cultured in CellGro stem cell growth media (SCGM; CellGenix, Freiburg, Germany), supplemented with recombinant human cytokines thrombopoietin, FltL, and stem cell factor at 100 ng/mL (CellGenix). CD34^+^ cells were thawed and prestimulated for 24–48 hr at 1 × 10^6^ cells/mL in cytokine-supplemented media as described above. Cells were then transduced with LVV in cytokine-supplemented media as described above and protamine sulfate at a final concentration of 8 μg/mL (APP Pharmaceuticals, Schaumburg, IL, USA) for 24 hr at 4 × 10^6^ cells/mL. Cells were then washed and maintained in cytokine-supplemented media for 3 days (GFP vector) or 7 days (Globin vector) prior to being subjected to flow cytometric analysis or qPCR for assessment of VCN. For methylcellulose colony-forming unit assays following lentiviral transduction, approximately 500 cells were plated into Methocult Classic H4434 (STEMCELL Technologies, Vancouver, BC, Canada). Following 12–14 days of culture, colonies were scored by morphology and either plucked as individual colonies or pooled and subjected to qPCR for assessment of VCN. Primary CD34^+^ cells from sickle cell disease and β-thalassemia patients were obtained from Hospital Mondor (Paris, France) for research use.

### HPLC

HPLC analyses were performed with a Prominence chromatograph (Shimadzu, Somerset, NJ, USA) and its LC Solution software. Globin chains from pooled erythroid colonies were separated by reverse-phase HPLC using a 4.6-mm Aeris 3.6-μM Widepore C4 Column (Phenomenex, Torrance, CA, USA). Samples were eluted with a gradient mixture of solution A (water with 0.1% trifluoroacetic acid) and solution B (acetonitrile with 0.08% trifluoroacetic acid). The absorbance was measured at 220 nm.

### PCR and VCN Assay

Genomic DNA was isolated from cell cultures using QIAGEN DNeasy protocol (QIAGEN, Hilden, Germany). PCR was performed using TaqMan Fast Master Mix (Invitrogen, Carlsbad, CA, USA) and 0.9 μM GAG forward (5′-GGAGCTAGAACGATTCGCAGTTA-3′) and reverse (5′-GGTTGTAGCTGTCCCAGTATTTGTC-3′) primers and GAG FAM probe [5′-(FAM)-ACAGCCTTCTGATGTCTCTAAAAGGCCAGG-(TAMRA)-3′] and RNASE-P-VIC control TaqMan assay (Invitrogen), and run using Fast program on Applied Biosystems StepOnePlus real-time thermocycler (Invitrogen). VCN was assessed relative to the Clone K3 cDNA, known to contain two copies of integrated viral DNA per cell.^2^

### Engraftment Assay

Female NOD-Cg-Prkdc^scid^Il2rg^tm1Wjl^/Sz (NSG) mice were conditioned with 30 mg/kg busulfan or by irradiation with 270 cGy (cesium source) at day −1 and then transplanted intravenously (i.v.) with 1E6 CD34^+^ cells. Mice were maintained in sterile conditions and provided with food and water ad libitum. At 4 months post-transplant, bone marrow from the femur was also collected. Cells were analyzed by flow cytometry for GFP, CD3, CD19, CD33, and huCD45. Additionally, bone marrow was processed for genomic DNA, VCN analysis, and integration-site analysis. All protocols were approved by local Institutional Animal Care and Use Committee (IACUC) (bluebird bio, Scripps Research Institute, and Toxicon).

### Flow Cytometry

Antibodies were purchased from BioLegend and from BD Biosciences. Flow cytometry was performed using an Accuri C6 (BD Biosciences, San Jose, CA, USA), a four-laser SORP BD Fortessa (BD Biosciences), or two-laser SORP BD Aria (BD Biosciences), and analysis was performed using FlowJo software (Tree Star, Ashland, OR, USA).

### Integration-Site Analysis

We followed the approach of Zhou et al.^29^ In brief, 1 μg of genomic DNA (gDNA) was sheared using Covaris sonicator, followed by end repair, A-tailing, adaptor ligation, and linear amplification-mediated (LAM) PCR with biotinylated primers. Cleaned up LAM products were further amplified using nested PCR with primers carrying sample-specific bar codes. The libraries were sequenced on Illumina NextSeq in a 150-cycle pair-end run. The reads were trimmed of viral sequences and aligned to hg38 reference genome using bowtie2.^30^ The positions of mapped reads were further annotated with information about corresponding gene models and features taken from the ENSEMBL genomes database.^31^

### Statistical Analysis

Our standard statistical test is a two-sided unpaired t test. Statistical significance is indicated with a p value; N.S. denotes p > 0.05.

## Author Contributions

G.C.H., M.B., L.C., F.J.P., A.H., S.S., G.L., and O.G. performed most of the experiments; G.C.H., M.B., L.C., F.J.P., D.C., Y.S., W.Z., and G.L. developed assays and analyzed samples; G.C.H., M.B., L.C., F.J.P., Y.S., W.Z., K.A.G., H.H., B.E.T., M.H.F., P.D.G., and G.V. designed the experiments and analyzed data; G.C.H. wrote the manuscript, which was reviewed by all authors.

## Conflicts of Interest

The following authors are or were full-time employees of bluebird bio, Inc. and receive salary and hold equity in bluebird bio, Inc.: G.C.H., M.B., L.C., F.J.P., D.C., Y.S., W.Z., A.H., S.S., G.L., K.A.G., H.H., M.H.F., P.D.G., and G.V. The following authors declare no potential conflict of interest: O.G. and B.E.T.

## Supplemental Information

Document S1. Figures S1–S5 and Table S1

Document S2. Article plus Supplemental Information
