# Supplementary material for: Prostaglandin E2 Increases Lentiviral Vector Transduction Efficiency of Adult Human Hematopoietic Stem and Progenitor Cells
Source: Mol Ther. 2017 Oct 5;26(1):320–8. doi: 10.1016/j.ymthe.2017.09.025 (PMC5763075; doi:10.1016/j.ymthe.2017.09.025)
Supplement: Document S1. Figures S1–S5 and Table S1 [file mmc1.pdf]

YMTHE, Volume *26*

Supplemental Information

Prostaglandin E_2 Increases Lentiviral Vector Transduction Efficiency of Adult Human Hematopoietic Stem and Progenitor Cells

Garrett C. Heffner, Melissa Bonner, Lauryn Christiansen, Francis J. Pierciey, Dakota Campbell, Yegor Smurnyy, Wenliang Zhang, Amanda Hamel, Seema Shaw, Gretchen Lewis, Kendrick A. Goss, Olivia Garijo, Bruce E. Torbett, Holly Horton, Mitchell H. Finer, Philip D. Gregory, and Gabor Veres

Heffner GC et al, Prostaglandin E_2 increases lentiviral vector transduction efficiency of adult human hematopoietic stem and progenitor cells

SUPPLEMENTARY APPENDIX

Figure S1. PGE_2 increases VCN over a broad range of MOI

CD34+ cells were transduced with research-grade LVV containing MND-ALDP transgene at MOI 4, 8, 12, 16, or 32 in the presence or absence of 10 µM PGE_2, and the vector copy number was determined following 14 days in a standard methylcellulose culture assay. Methylcellulose cultures were performed in triplicate, and a VCN was determined for each replicate culture.

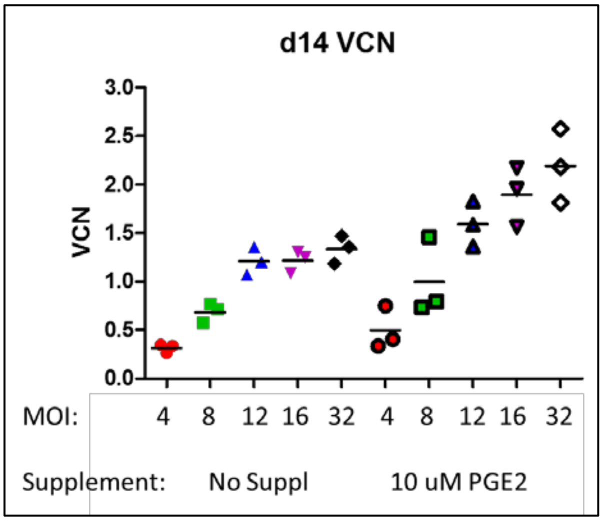

Figure S2. Comparable viability and cell count following transduction of CD34+ cells in the presence or absence of 10 µM PGE_2

CD34+ cells were transduced with LVV in the presence or absence of 10 µM PGE2 for 24 hours, and the D1 viability (by trypan blue exclusion) and the cell counts were determined, and compared to mock-transduced cells. Representative data from six independent experiments are shown.

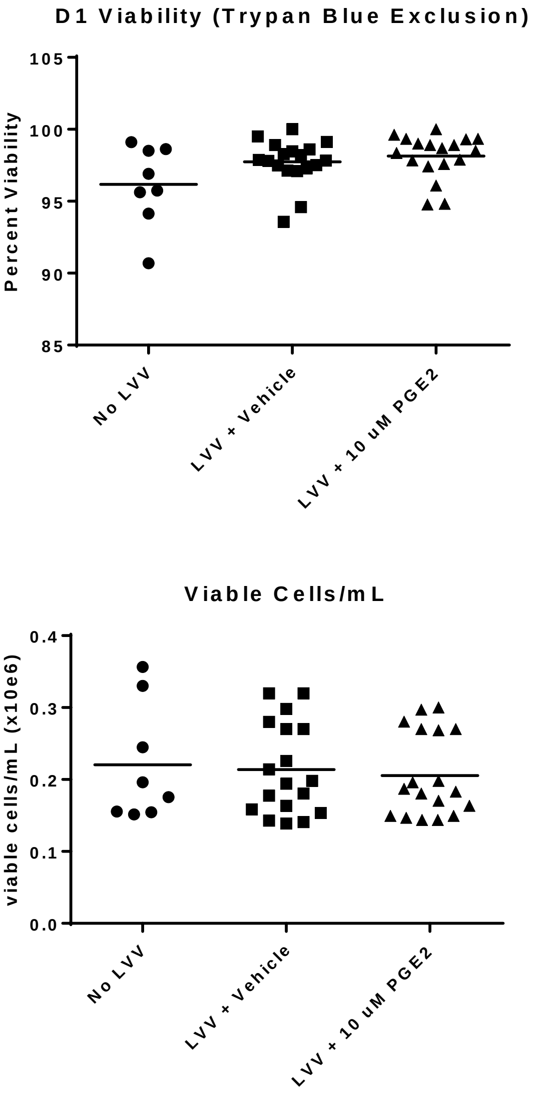

Figure S3. PGE_2 does not promote viral entry by BlaM assay

Beta lactamase assay using VPR-BlaM loaded LVV particles was performed as previously described (Cavrois et al., 2002) using GeneBLAzer cell-based assay (Invitrogen, Carlsbad, CA). CD34+ cells were exposed to a VPR-BlaM LVV and analyzed by flow cytometry for BlaM activity. Representative FACS plot depicting a No-Envelope control vector (a), or VSV-G envelope vector in the presence of vehicle (b), or 10 µM PGE_2 (c). The percentage of BlaM+ cells for triplicate samples are summarized in (d). LVV, lentiviral vector; PGE_2, prostaglandin E2; BlaM, Beta-lactamase.

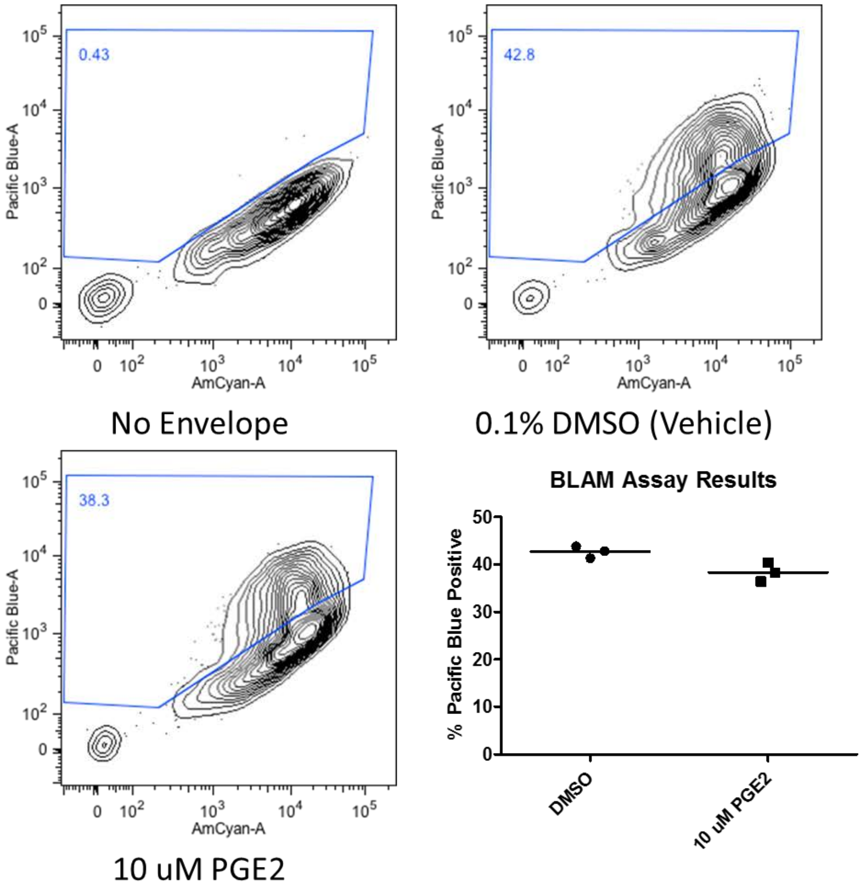

Figure S4. PGE_2 improves transduction of CD34+ CD38- cells in culture

CD34+ CD38- cells were sorted on a BD FACSAria II. Transduction of CD34+ cells and CD34+ CD38- cells with LVV supplemented with 10 µM of PGE_2 as indicated. The mean and VCN is indicated for triplicate wells per condition, as assessed at Day 14 post-transduction. LVV, lentiviral vector; PGE_2, prostaglandin E2; VCN, vector copy number.

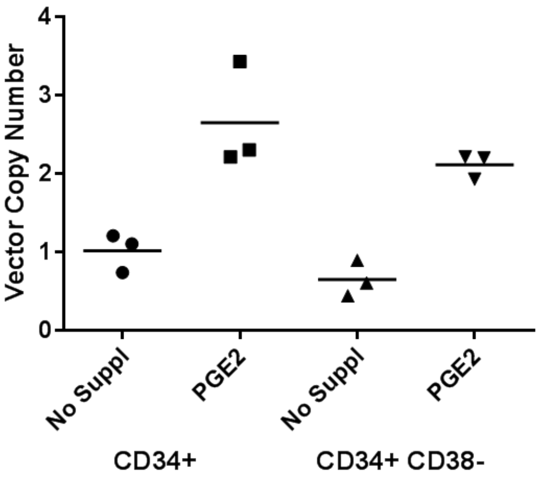

Figure S5. Similar engraftment and lineage outputs of mock- and vehicle-exposed CD34+ cells

CD34+ cells were transplanted following culture in the presence or absence of 0.1% DMSO during *ex vivo* lentiviral transduction. Representative results depicting huCD45+ chimerism, huCD3+ chimerism, huCD19+ chimerism, and huCD33+ chimerism are depicted for three independent transplant experiments.

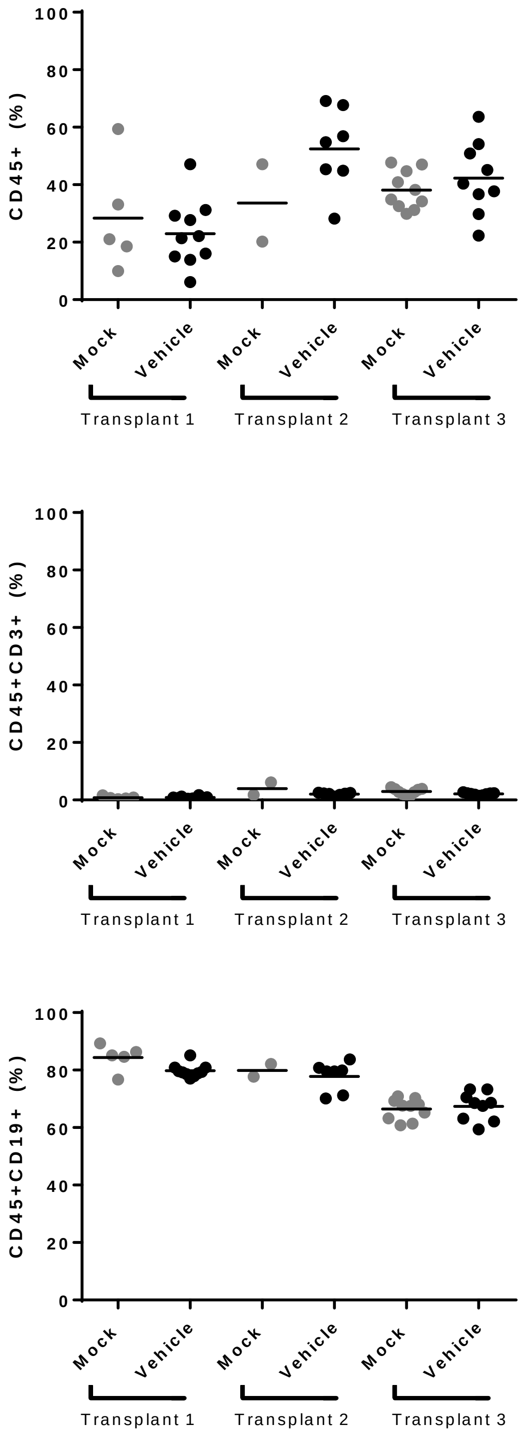

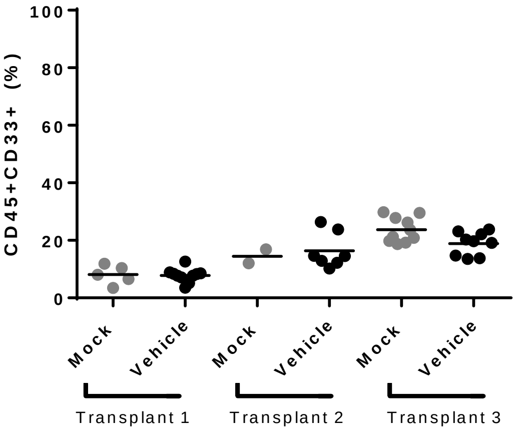

CD45+CD33+ (%)
100
80
60
40
20
0
Mock
Vehicle
Mock
Vehicle
Mock
Vehicle
Transplant 1
Transplant 2
Transplant 3

Table S1. PGE2 does not impact distribution of insertion sites among tumor suppressor or oncogenes

After completion of integration site analysis for each engrafted mouse, we annotated each insertion site as being within 30kb of either oncogene, tumor suppressor or neither. The lists of tumor suppressor and oncogenes were obtained from UniProt database (PMID 25348405). We calculated the percentages of reads found at tumor suppressor and oncogene loci for each animal and used one-way ANOVA to test for statistical significance between DMSO and PGE_2-treated animals. The corresponding p-values were p=0.87 for oncogenes and p=0.16 for tumor suppressors.

Number of reads from all samples				Total
Condition	oncogene	tumor suppressor	neither	
DMSO	7222	5534	301133	313889
PGE2	5065	1893	307500	314458
**Percentages of reads from genes in each category**				
**Condition**	**Oncogene**	**Tumor Suppressor**	**Neither**	
DMSO	2.30%	1.76%	95.94%	
PGE2	1.61%	0.60%	97.79%	
